# Implicit Mentalising during Level-1 Visual Perspective-Taking Indicated by Dissociation with Attention Orienting

**DOI:** 10.3390/vision2010003

**Published:** 2018-01-20

**Authors:** Mark R. Gardner, Aiste P. Bileviciute, Caroline J. Edmonds

**Affiliations:** 1 Department of Psychology, University of Westminster, 115 New Cavendish Street, London W1W 6UW, UK; 2 School of Psychology, University of East London, Stratford Campus, Water Lane, London E15 7LZ, UK

**Keywords:** social attention, attention orienting, visual perspective-taking, Theory of Mind, implicit mentalising

## Abstract

Experiments demonstrating level-1 visual perspective-taking have been interpreted as providing important evidence for ‘implicit mentalising’—the ability to track simple mental states in a fast and efficient manner. However, this interpretation has been contested by a rival ‘submentalising’ account that proposes that these experiments can be explained by the general purpose mechanisms responsible for attentional orienting. Here, we aim to discriminate between these competing accounts by examining whether a gaze aversion manipulation expected to enhance attention orienting would have similar effects on both perspective-taking and attention orienting tasks. Gaze aversion was operationalised by manipulating head position relative to torso of the avatar figures employed in two experiments (gaze-averted vs. gaze-maintained). Experiment 1 used a Posner cueing task to establish that gaze aversion enhanced attention orienting cued by these avatars. Using the avatar task, Experiment 2 revealed level-1 visual perspective-taking effects of equivalent magnitude for gaze-averted and gaze-maintained conditions. These results indicate that gaze aversion moderated attention orienting but not perspective-taking. This dissociation in performance favours implicit mentalising by casting doubt on the submentalising account. It further constrains theorising by implying that attention orienting is not integral to the system permitting the relatively automatic tracking of mental states.

## 1. Introduction

Understanding what other people know—an ability often called mentalising or perspective-taking—is crucial to effective social interaction. A distinction has been drawn between a system that enables *automatic* tracking of mental states and an effortful system that enables these states to be explicitly reasoned about (e.g., [1,2]). There is currently intense debate about whether the automatic system is specialised (mentalising position: e.g., [1,3]), or mediated by general-purpose cognitive processes (submentalising position: e.g., [4]). Resolving this issue is essential to determine whether there are one or two dedicated systems for Theory of Mind [5,6], with implications for understanding Autism Spectrum disorders [7,8]. This paper addresses this issue by distinguishing between submentalising and mentalising accounts for automatic level 1 visual perspective-taking (hereafter, L1-VPT).

The *avatar task* introduced by Samson et al. [9] provides important evidence of L1-VPT. Participants identify the number of items seen either from their own perspective (‘Self perspective’ trials) or from that of an avatar (‘Other perspective’ trials), while the avatar is orientated towards either a matching or a non-matching number of items. Performance during both types of trials is poorer for non-matching compared to matching trials (e.g., [9,10]), referred to as a ‘consistency effect’. The consistency effect for the ‘Self perspective’ trials is particular pertinent for the measurement of L1-VPT because it appears to indicate that another person’s mental states have been tracked and intrude or interfere with performance from one’s own perspective. The cognitive process captured by this consistency effect satisfies most criteria of automaticity [11]. The effect is unintentional, and occurs despite the avatar being formally task irrelevant. It is efficient/effortless; the effect is neither affected by concurrent task demands [12], nor time pressure [13]. It is not, however, mandatorily triggered by the mere presence of an avatar—indicated by the effect being moderated by, or contingent upon, the perspective-taking context [14,15,16,17]. Thus, an intuitively appealing account of these findings is that the avatar’s head and/or body orientation enables relatively automatic computation of *what is seen* by the avatar, perhaps via line-of-sight computation [18].

A submentalising account proposes that domain general processes may explain the consistency effect, without recourse to ascription of mental states [4]. Specifically, the avatar’s head and/or body orientation might spatially orient participants’ attention, leading to enhanced performance when this region of space contains all the items visible to participants (matching trials). In line with this hypothesis, consistency effects of comparable magnitude occur when the avatar is replaced by an arrow ([19]; but see [20,21]). Furthermore, spatial cueing experiments have revealed that the original avatar stimuli elicit shifts of attention towards the direction faced by the avatar cue when it preceded the target by a stimulus onset asynchrony (SOA) of at least 300 ms [14] or 600 ms [17], but not 100 ms [15,17]. Cue-validity effects have occurred with simultaneous presentation of avatar and target only if instructions specifically encourage endogenous attention orienting [14]. Thus, avatars appear to engage voluntary, rather than reflexive, attention orienting, suggesting that volitional attention orienting could account for performance in the avatar task to the extent that the context promotes top-down modulation of such effects.

Several experiments have attempted to discriminate between these accounts by assessing predictions of the mentalising account [3,22,23,24]. Cole et al. [22] introduced transparent or opaque barriers in front of the avatar, and found consistency effects for both an opaque and transparent condition, contrary to mentalising predictions. By contrast, other experiments examining the effects of barriers with and without apertures found consistency effects contingent upon an unbroken line-of-sight [23]. A related tactic is to manipulate participants’ beliefs about whether the avatar could see the items [4,25]. Furlanetto et al. [3] found a consistency effect restricted to avatars wearing goggles of a colour believed by participants to be transparent (and not for an alternative colour believed to be opaque), offering support to the implicit mentalising account. However, the selectivity of this effect was not replicated by Conway et al. [24], nor Wilson et al. for blindfolded avatars [26]. Thus, research seeking to evaluate the mentalising account is inconclusive.

In the present study, our innovation is to test predictions of the submentalising account rather than the mentalising account. In contrast to previous work, our approach is to evaluate whether a manipulation expected to influence attention orienting, but not mentalising, would influence VPT-1. Crucially, we also assess the effect of the same manipulation on a conventional attention orienting task, to determine whether it has a differential effect on VPT-1 and attention orienting.

**Figure 1:**
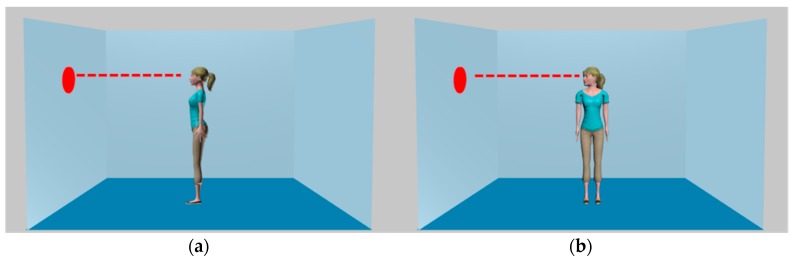
Gaze-maintained (**a**) and gaze-averted (**b**) avatars used in Experiments 1 and 2, illustrating that there is unbroken light of sight between the target dot and the avatar in both cases (right facing avatars not illustrated).

The manipulation chosen for this purpose was Avatar-Stance (see Figure 1). Spatial cueing experiments have established that head orientation directs attention [27], more than body orientation [28]. These directional cues interact, with directional cueing being stronger for body stances in which head orientation is not aligned with the torso (e.g., the gaze-averted stimulus in Figure 1), than when head and torso are aligned (e.g., gaze-maintained stimulus) [29,30]. It has been suggested that the gaze-averted stimulus provides an enhanced cueing effect, because this combination more reliably indicates a person’s active attentional behaviour than the more passive gaze-maintained combination [29]. Nonetheless, both types of stimuli have an unbroken line-of-sight and equivalent visual access to the target. We therefore adopted Avatar-Stance as a manipulation expected to influence consistency effects driven by attention orienting, but not mentalising.

Two experiments utilised the avatars illustrated in Figure 1. The objective of Experiment 1 was simply to confirm that Avatar-Stance moderates attention orienting elicited by these avatars. This employed a standard attention orienting paradigm, a Posner detection task, in line with our previous work [17]. Attention orienting is indicated by shorter RTs to detect the appearance of a target when an avatar cue is directed towards the target (valid trial) than when the avatar is directed away from the target (invalid trial). An enhanced cue-validity effect for gaze-averted characters was predicted, based on previous findings for photographic stimuli [29]. Additionally, manipulating the SOA between cue and target enabled assessment of reflexive (short SOA) as well as volitional attention orienting (cue validity effect at long SOA). Thus, a fully within-participants design was employed in which the factors were Validity, Avatar-Stance, and SOA.

Experiment 2 employed the avatar task to examine whether the Avatar-Stance manipulation would have similar effects in a perspective-taking context, and when avatar and target were presented simultaneously (SOA = 0 ms). Participants judged the number of dots from their own perspective (‘self perspective’ trials), with both Avatar-Stance and Consistency (of the number of dots seen by participant and avatar) manipulated within participant. The submentalising account predicts that this manipulation would affect perspective-taking similarly—specifically, a stronger consistency effect would be expected for gaze-averted compared with gaze-maintained stimuli. By contrast, the mentalising account predicts a consistency effect that is not moderated by Avatar-Stance. This is because for both stances, there is an unbroken line-of-sight to the items, with equivalent visual access.

## 2. Materials and Methods 

### 2.1. Experiment 1 (Posner Task)

#### 2.1.1. Participants

Members of the University of Westminster community (*N* = 32, 21 female) participated for an honorarium (£10), after providing informed consent. The study was conducted in accordance with the Declaration of Helsinki, and the protocol was approved by the University of Westminster Ethics Committee (VRE1516-0072). Ages ranged from 18 to 33 years (*M* = 21.6, *SD* = 2.88). This sample size was chosen to correspond to the planned sample size of Experiment 2 and our earlier work demonstrating cue-validity effects for avatar figures using a similar design [17].

#### 2.1.2. Stimuli and Procedure

Stimuli, illustrated in Figure 1, consisted of a female avatar provided by mixamo.com, digitally edited into the virtual rooms employed by Samson et al. [9]. Although the original task matched the gender of the avatar to the participants, we employed the same female avatars throughout because the population we sampled from was predominately female, and the authors of the original task [9] suggest that gender matching is not necessary. For gaze-maintained avatars, both torso and head were oriented to the side, in keeping with the original avatar task. For gaze-averted avatars, the torso faced the participant, while the head was oriented to the side. Stimulus presentation and data collection were controlled by E-Prime [31], running on a Dell PC with 22-inch screen.

In the trials, a fixation cross was superimposed on the virtual room. It was displayed for 750 ms, and replaced after 500 ms by the avatar facing left or right. The target (dot) was then presented with a variable delay (SOA = 100, 300, 600 ms). These stimuli were displayed for a maximum of 3000 ms or until a key was pressed. During inter-trial intervals (500 ms) the room continued to be presented.

Participants first completed a short practice block (14 trials), followed by four experimental blocks. Each block included 80 trials, comprising 72 target present and 8 catch trials in which no target was presented. Trials were randomly ordered, with all combinations of Avatar-Stance, SOA, cue direction, and cue validity occurring on 3 trials within each block. Participants were instructed to press the response key (“H”) immediately on detecting the target, and not to respond if no target was presented. Participants were told that avatars did not predict target location and to maintain fixation at centre of the screen.

### 2.2. Experiment 2 (Avatar Task)

#### 2.2.1. Participants

In total, 35 volunteers who had not participated in Experiment 1, but were drawn from the same population, participated for an honorarium (£10) after providing consent. One participant who made a high number of errors was excluded (see Section 2.2), resulting in a sample size of 34 (20 female), aged 20 to 63 years (*M* = 28.8, *SD* = 10.7). This sample exceeds the 24 participants required to detect the effect size obtained by Samson et al. for the consistency effect ([9], Experiment 3, simple effect when the central stimulus was an avatar), *d* = 0.61, with 80% power. We also slightly exceeded our target sample size of 32, based on our earlier work [17], due to over-booking on the final day of testing.

#### 2.2.2. Stimuli and Procedure

Stimuli were the same avatars as Experiment 1, presented within the virtual room, always oriented either to the left or right (illustrated in Figure 1). A variable number of dots were presented (0–3), which appeared on the left and/or right walls, corresponding to the distributions employed in previous work (e.g., [9,19]). Data collection used the same computer as Experiment 1. “Yes” responses were made by pressing the “J” key, and “No” responses by pressing the “K” key.

Trials began with the presentation of a fixation cross for 750 ms. After 500 ms, the prompt “YOU” was displayed for 750 ms. Following another 500 ms interval, a digit (0–3) was presented for 750 ms. The virtual room was then presented with both the avatar and 0–3 dots, for a maximum of 2000 ms or until a response was detected. Participants were instructed to respond “Yes” or “No” to whether the digit presented corresponded to the number of dots seen from their own perspective, as quickly and accurately as possible. 

The experiment comprised 208 trials, including 16 filler trials in which no dots were presented. Trials were randomly ordered, with 48 for each combination of Avatar-Stance and Consistency (number of dots seen by avatar and participant are consistent/inconsistent). Within each condition, yes and no responses, and direction of avatar orientation (left/right) were equiprobable using the original trial compositions [9]. These trials were distributed across 4 blocks (52 trials) preceded by a practice block (26 trials).

## 3. Results and Discussion

### 3.1. Experiment 1 (Posner Task)

Rates of false alarms during catch trials were low (*M* = 2.9%, *SD* = 4.1). Mean RT was computed for each participant and condition, excluding catch trials and response omissions through timeout (0.07% of the data).

**Figure 2:**
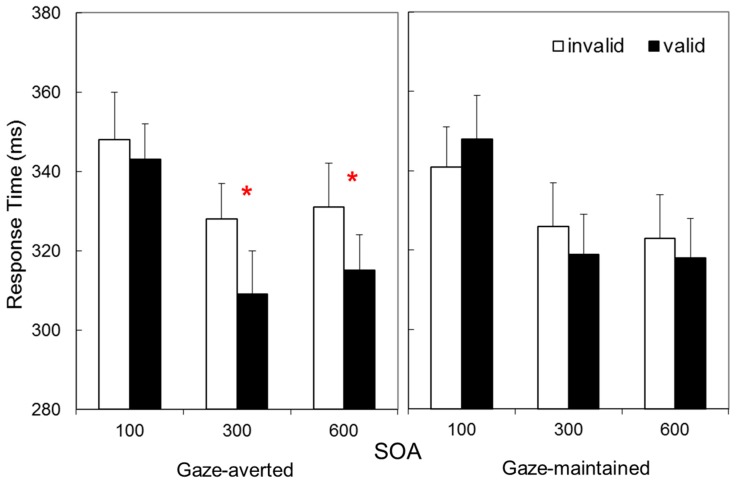
Data from Experiment 1. Response times in a Posner task as a function of whether an avatar cue was gaze-averted or gaze-maintained, and whether it was a valid or invalid predictor of a target at three cue-target onset asynchronies. * Indicates statistically significant simple effect, *p* < 0.001.

Figure 2 presents Mean RT as a function of validity, SOA, and Avatar-Stance. These data suggest a cue validity effect only for gaze-averted avatars at longer SOAs. These impressions were confirmed by a 2 × 3 × 2 repeated measures Analysis of Variance (ANOVA), with Validity (valid vs. invalid), SOA (100 vs. 300 vs. 600 ms), and Avatar-Stance (gaze-averted vs. gaze-maintained) as factors. This revealed main effects of Validity, *F*(1, 31) = 10.9, *p* = 0.002, η_p_^2^ = 0.26, indicating shorter RTs for valid than invalid trials, and SOA, *F*(2, 62) = 40.6, *p* < 0.001, η_p_^2^ = 0.57, consistent with longer RTs at short SOA, but not Avatar-Stance, *F* (1, 31) = 0.002, *p* = 0.962, η_p_^2^ = 0.00. Crucially, Validity interacted with Avatar-Stance, *F*(1, 31) = 16.2, *p* < 0.001, η_p_^2^ = 0.34, as well as SOA, *F*(2, 62) = 3.99, *p* = 0.023, η_p_^2^ = 0.11. The three-way interaction was not statistically significant, *F* (2, 62) = 0.013, *p* = 0.987, η_p_^2^ = 0.00.

These interactions were examined with a Validity × SOA ANOVA for each Avatar-Stance. These confirmed that a main effect of Validity was present for gaze-averted avatars, *F*(1, 31) = 32.7, *p* < 0.001, η_p_^2^ = 0.51, but not for gaze-maintained, *F*(1, 31) = 0.2, *p* = 0.627, η_p_^2^ = 0.01. Related *t*-tests on the gaze averted data indicated statistically significant cue validity effects at 300 ms SOA, *M* ± *SD* = 19 ± 25 ms, *t*(31) = 4.33, *p* < 0.001, and 600 ms SOA, 16 ± 27 ms, *t*(31) = 3.49, *p* = 0.001 (100 ms: 5 ± 23, *t*(31) = 1.09, *p* = 0.284).

Experiment 1 thus provides a replication of the finding that attention orienting is more likely to occur when head-orientation is averted relative to the torso [29], and extends this finding from photographs to avatar stimuli. The finding that attention orienting was present only for longer SOAs is consistent with previous experiments examining avatars as spatial cues [14,15,17], and is indicative of volitional rather than reflexive attention orienting. However, the absence of an effect for gaze-maintained stimuli in the present study contrasts with positive effects previously reported [14,17]. This might have been an unanticipated consequence of differences in the low-level features of the different avatar character employed. Nonetheless, the interaction between Avatar-Stance and Validity confirmed that attention orienting for the new characters employed in the present study is more likely to occur when head-orientation is averted relative to the torso.

Having established a manipulation that affects attention orienting for these stimuli, the purpose of Experiment 2 was to assess whether Gaze-Aversion similarly moderates L1-VPT.

### 3.2. Experiment 2 (Avatar Task)

Data were excluded for “no” responses (number of dots did not match the digit), and “filler” trials (no dots present). We computed Percentage of Errors (PE) and mean Response Times (RTs) for correct responses. One participant was excluded due to high error rate (PE = 12%) relative to the remaining sample (*N* = 34, *M* = 3.0%, *SD* = 2.77%). There were minimal response omissions due to timeout (0.2%).

**Figure 3:**
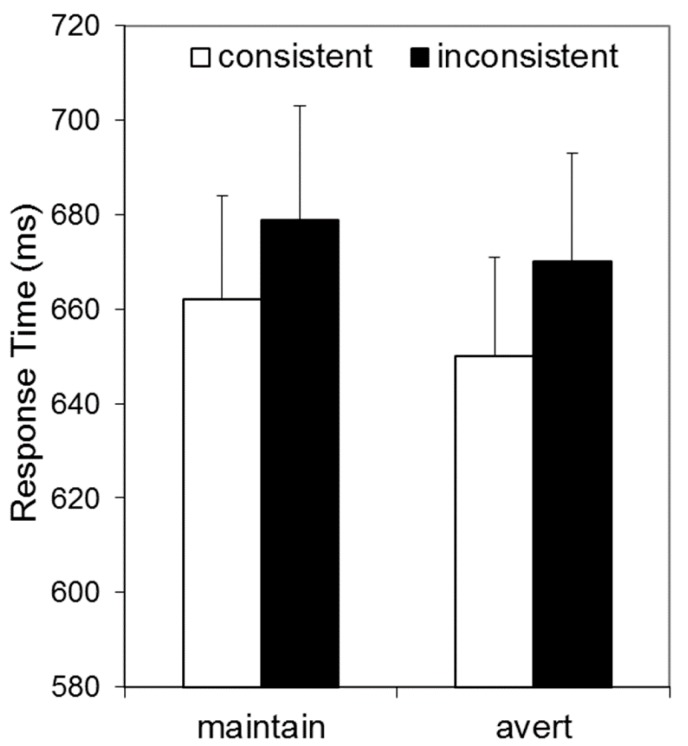
Data from Experiment 2. Response times in an adapted Samson avatar task as a function of whether the number of dots visible to the avatar and participant were consistent, and whether avatar gaze was maintained or averted.

Figure 3 illustrates that RTs appeared to be elevated for inconsistent relative to consistent conditions, and by a similar extent for gaze-averted and gaze-maintained avatars. These data were analysed by a 2 × 2 repeated measures ANOVA, with Consistency (consistent vs. inconsistent) and Avatar-Stance (gaze-averted vs. gaze-maintained) as factors. This revealed a main effect of Consistency, *F*(1, 33) = 5.72, *p* = 0.023, η_p_^2^ = 0.15, confirming that RTs were longer for inconsistent than consistent trials, and no effect of Avatar-Stance, *F*(1, 33) = 2.84, *p* = 0.101, η_p_^2^ = 0.08. Crucially, there was no interaction between these factors, *F*(1, 33) = 0.08, *p* = 0.783, η_p_^2^ = 0.00.

A Bayesian approach was employed to examine further the absence of an interaction between Consistency and Avatar-Stance. Bayesian statistics can determine the relative support for the null hypothesis (no interaction, as predicted by the mentalising account) versus the alternative hypothesis (interaction, as predicted by the submentalising account), thus addressing limitations of the null hypothesis significance test approach when *p* is non significant. A Bayes Factor of 1 indicates that the null and alterative hypothesis are equally likely, given the obtained data; whereas, by convention, values below 1/3 are taken as evidence in favour of the alternative hypothesis, and values above 3 are taken as evidence in favour of the null hypothesis. Using the method described by Masson [32], the Bayes Factor computed for the Consistency x Avatar-Stance interaction was found to favour the null, BF01 = 8.96. The posterior probability for the null hypothesis, *p*_BIC_(H_0_|D) = 0.90, and for the alternative hypothesis, *p*_BIC_(H_1_|D) = 0.10, provide positive evidence favouring the null hypothesis, based on the classification provided by Raftery [33].

Analyses of PE confirmed that a speed-accuracy trade-off had not concealed an effect of Avatar-Stance. There were neither main effects of Consistency, *F*(1, 33) = 0.51, *p* = 0.481, η_p_^2^ = 0.02, nor Avatar-Stance, *F*(1, 33) = 1.59, *p* = 0.216, η_p_^2^ = 0.05, and crucially no interaction, *F*(1, 33) = 0.32, *p* = 0.578, η_p_^2^ = 0.01.

Therefore, Experiment 2 provided evidence of L1-VPT in the avatar task by showing that RTs were longer when the number of items visible to the avatar and the participant were inconsistent than when they were consistent. This consistency effect is in line with many previous experiments employing the avatar task (e.g., [9,19,22,24]).

Crucially, Experiment 2 found that the magnitude of this consistency effect was not moderated by avatar stance, a manipulation that facilitates attention orientation for these stimuli. It is important to stress that in the avatar task it is this consistency effect which is the putative measure of L1-VPT, not response times *per se*. Thus, a main effect of Avatar-Stance would have indicated that Avatar-Stance influences participants’ ability to confirm the number of dots presented (seen from their own perspective) as quickly and as accurately as possible. This involves subitizing items, and comparing this quantity to a number held in working memory. It does not involve perspective-taking. In fact, explicit perspective-taking was never required in Experiment 2, because there were no ‘other perspective’ trials, and the avatar is formally task irrelevant. If we had found that response times were generally shorter for the gaze-averted condition (main effect of Avatar-Stance—non-signficant in the current experiement), this would have merely indicated that these stimuli provide less interference than gaze-maintained stimuli for the processes involved in confirming the number of dots present (subitizing and comparison of this quantity to a value held in working memory). Whereas, the absence of a Consistency x Avatar-Stance interaction is the crucial finding in Experiment 2, because this uniquely relates to L1-VPT, automatic level 1 visual perspective-taking.

## 4. General Discussion

We sought to test a submentalising account of level-1 visual perspective-taking (L1-VPT) by assessing whether a manipulation that affected attention orienting had a similar effect on L1-VPT. Experiment 1 revealed that gaze-averted avatars orient attention to a greater extent than gaze-maintained avatars. Experiment 2 demonstrated that this manipulation did not affect L1-VPT, by revealing a consistency effect in the avatar task that was not moderated by avatar stance. Therefore, the present study demonstrates that conditions that facilitate attention orienting do not facilitate L1-VPT as measured by the avatar task. This dissociation between spatial cueing and perspective-taking tasks is inconsistent with predictions of the submentalising account, thus lending support to the mentalising account.

The finding that a SOA of at least 300 ms was required for avatars to elicit attention orienting lends further support to the view that body orientation supports voluntary, and not reflexive, attention orienting [14,15,17]. For L1-VPT measured by the avatar task, this would imply that volitional attention orienting may contribute to performance to the extent that the perspective-taking context promotes top-down modulation of such effects [15,17]. At first glance, it might appear that such an account is unsuitable, given that the avatar task involves simultaneous presentation of the avatar and the target/dot, in contrast to typical attention orienting tasks. However, Bukowski et al. [14] have shown that cue-validity effects for avatars may occur even with simultaneous presentation of avatar and target if instructions specifically encourage endogenous attention orienting. Nonetheless, the present evidence for a dissociation between perspective-taking and attention orienting suggests that typical avatar task instructions do not promote use of this slow-acting attention orienting process when social cue and target are simultaneously available.

This dissociation may also constrain theorising about the nature of implicit mentalising in L1-VPT. Mentalising accounts have yet to specify how what is seen by the avatar is automatically computed, remaining agnostic regarding whether attention orienting is involved [3,9]. Our finding that Avatar-Stance did not influence performance in the avatar task suggests that attention orienting may not be integral to the tracking of these mental states. This finding therefore encourages further investigation of the role of line-of-sight computation for automatic processing of what others see [18,23].

In relation to social attention, Experiment 1 revealed that gaze-averted avatars—in which the torso faces participants—were a more effective directional cue than gaze-maintained avatars. This effect replicates earlier findings using photographic stimuli [29]. These findings are consistent with the intrinsic representation of spatial codes [34,35], and evidence that head orientation is a more powerful cue than body orientation [28]. It has been proposed that stronger attention orienting effects for gaze-averted stimuli may occur because this is an active rather than a passive stance, and thus provides more informative about where another person is attending [29,36]. Relatedly, photographs of left/right-facing bodies have been found to elicit attention orienting when the photographs implied that the actor was moving, but not when the actor was passively standing [37]. These phenomena warrant further investigation.

In conclusion, the present study demonstrates a dissociation between performance of L1-VPT and Posner tasks. This dissociation has three main implications. Firstly, it undermines the current submentalising account for L1-VPT based upon attention orienting by showing that conditions that facilitate attention orienting do not facilitate L1-VPT. Secondly, it encourages further investigation into how social attention is influenced by the stance as well as orientation of the body. Thirdly, it suggests that attention orienting is not integral to the system, permitting relatively automatic tracking of mental states. We suggest that a perspective-taking context and/or simultaneous availability of the social cue and target limits the influence of attention orienting. By undermining this particular submentalising account, our findings lend support to the mentalising account that proposes that L1-VPT in the avatar task is the relatively automatic computation of what is seen by the avatar.
